# Supplementary material for: Effect of neostigmine/glycopyrrolate versus sugammadex on postoperative delirium in older adults: A triple-masked, randomized, controlled trial protocol
Source: PLoS One. 2026 Apr 1;21(4):e0346523. doi: 10.1371/journal.pone.0346523 (PMC13042718; doi:10.1371/journal.pone.0346523)
Supplement: S1 File — (PDF) [file pone.0346523.s001.pdf]

研究方案

版本号：1.0

版本日期： 2024 年 10 月 25 日

一、研究项目名称

新斯的明/格隆溴铵对比舒更葡糖对老年患者非心脏大手术后谵妄的影响：一项随机对照研究

二、研究背景

术后谵妄（postoperative delirium, POD）是一组表现为注意力下降、意识水平波动、认知功能障碍的临床急性脑综合征，是老年人最多见的术后并发症之一。根据 2018 年修订的术后认知功能障碍的概念，POD 是指术后 1 周内发生的急性认知功能受损[1]。在不同风险的手术后，POD 的患病率也不同，而在行非心脏大手术的老年人中，POD 的患病率为 20%~30%[2]。POD 给患者术后恢复带来了很多阻碍，如住院时间的延长、其他并发症发生风险的上升、医疗成本的增加以及死亡率的增加等。POD 的病因主要包括中枢神经的系统损伤（缺氧、低血糖、代谢紊乱等）及异常的应激反应（全身炎症反应等），而这两种机制在老年人的大脑中影响更为明显[3]，从而导致了老年人术后 POD 患病率更高。POD 的主要风险因素包括高龄、麻醉时长增加、受教育程度低、二次手术、感染及呼吸系统并发症等[4]。

麻醉药物也与 POD 的发生有关[5]，肌松拮抗剂是其中之一。目前常用的肌松拮抗剂为舒更葡糖与新斯的明，二者作用机制并不相同。舒更葡糖是一种经修饰的γ-环糊精，与神经肌肉阻滞剂罗库溴铵或维库溴铵形成复合物，降低在神经肌肉接头处与烟碱受体结合的神经肌肉阻滞剂的数量，从而逆转罗库溴铵或维库溴铵诱导的神经肌肉阻滞，该药本身不影响乙酰胆碱在神经肌肉接头处的功能[6, 7]。新斯的明作为一种胆碱酯酶抑制剂，增加乙酰胆碱浓度，起到拮抗非去极化肌肉松弛药的残留肌松作用。而胆碱酯酶抑制剂增加突触间隙乙酰胆碱含量也是改善痴呆和认知功能最主要的作用机制，该类药物（如多奈哌齐、卡巴拉汀）是现今治疗轻、中度阿尔兹海默症的一线治疗药物。就新斯的明而言，当前研究表明该药有可能改善术后认知[8-11]。同时，为对抗新斯的明引起的毒蕈碱样作用，通常联合伍用抗胆碱类药。其中，格隆溴铵为季铵类抗胆碱药，不易透过血脑屏障等脂膜，与叔铵类抗胆碱药阿托品和氢溴酸东莨菪碱相比，格隆溴铵的中枢神经系统相关不良反应也更少[12, 13]。然而，目前关于新斯的明/格隆溴铵与舒更葡糖拮抗全麻后肌松对老年患者术后认知功能的研究仍有限。

因此，本课题拟开展一项前瞻性随机对照的临床研究，通过手术结束后使用新斯的明/格隆溴铵或舒更葡糖拮抗神经肌肉阻滞，评估对非心脏大手术老年患者术后 POD 发生率（主要结局）及其严重程度、持续时间、术后疼痛等（次要结局）的影响，为非心脏大手术老年患者围术期麻醉用药管理提供依据，促进围术期恢复质量的改善。

三、研究目的

评估新斯的明/格隆溴铵拮抗神经肌肉阻滞能否减少非心脏大手术老年患者术后谵妄的发生率, 从而为改善患者预后提供更优的麻醉用药策略，提高患者康复质量。

四、研究设计（包括研究的总体设计、样本量、参研单位数量、研究步骤和研究时限等）

1、总体设计

本研究是一个由研究者发起、单中心、前瞻性、随机对照、临床研究。

2、样本量计算

非心脏大手术老年患者术后谵妄的发生率约 20%~30%，我们假设舒更葡糖组 POD 发生率为 25%，同时结合最新研究，新斯的明能够改善术后认知功能达 50%，使用 PASS 15 软件（NCSS, LLC. Kaysville, Utah, USA）进行样本量计算，采用双侧检验 α 为 0.05，效应量为 80%，共需 304 例样本量，考虑 5%的失访率，计划纳入 320 例患者，各组为 160 例。

Two Independent Proportions (Null Case) Power Analysis

Numeric Results of Tests Based on the Difference: P1 - P2

H0: P1-P2=0. H1: P1-P2=D1<>0. Test Statistic: Z test with pooled variance

Power	Sample Size Grp 1 N1	Sample Size Grp 2 N2	Prop|H1 Grp 1 or Trtmnt P1	Prop Grp 2 or Control P2	Diff if H0 D0	Diff if H1 D1	Target Alpha	Actual Alpha	Beta
0.8003	152	152	0.1250	0.2500	0.0000	-0.1250	0.0500		0.1997

Note: exact results based on the binomial were only calculated when both N1 and N2 were less than 100.

3、参研单位

苏州大学附属第一医院

4、伦理和注册

获得伦理申请审批后，将在 WHO 一级注册机构中国临床试验注册中心进行在线注册并取得临床试验注册号。临床试验注册在入组第一例患者前完成。所有入组的患者均充分告知本研究的过程，并签署书面知情同意书。

5、研究步骤和时限

伦理注册后，开始按照方案计划初筛患者，签订知情同意书，按照电脑生成的随机列表进行入组，实施麻醉与手术，术后在规定的时间点进行访视，直至该患者出院。该研究预计

在 1.5 年内完成。

五、研究人群（包括纳入标准、排除标准、退出标准、终止标准等）

1、纳入标准

1）年龄≥65 岁，性别不限；

2）美国麻醉医师协会（American society of Anesthesiologists, ASA）分级 I-III 级；

3）行择期非心脏非神经大手术（胸科手术、腹部大手术、泌尿外科手术、四肢关节手术和脊柱外科手术），预计手术时间 ≥1.5 小时；

4）术后计划拔除气管导管回外科病房且预计术后住院天数 ≥2 天；

5）清楚了解研究过程并自愿参加，签署知情同意书。

2、排除标准

1）对研究中的药物过敏；

2）存在新斯的明禁忌征（癫痫、肠梗阻、泌尿道梗阻、支气管哮喘、青光眼）、瘫痪或神经肌肉疾病；

3）严重肝功能不全（Chil-Pugh C 级），肾功能衰竭；

4）长期使用胆碱酯酶抑制剂、抗胆碱能药物或精神类药物；

5）不能进行有效沟通或拒绝参与研究。

3、退出或中止标准：

1）术中出现严重不良反应（如危及生命的大出血、过敏性休克等）、二次手术；2）术后带管转入重症监护室；3）受试者或代理人撤回知情同意书。

六、研究实施的干预方案

术前一天访视病人，签署麻醉及科研项目的知情同意书，向病人说明相关评分量表的使用方法并进行基本资料收集（年龄、性别、受教育程度、BMI、合并症）及简易智力状态检查（MMSE）评分。手术当天不用术前用药，术前禁食 6 小时。患者入手术室后的标准监测包括：心电图（ECG），脉搏氧饱和度（SpO2），无创血压（NIBP），BIS 监测麻醉深度，肌松监测。完成外周静脉穿刺置管，并开始基础液体输注（平衡液，5 mL/kg/h）。患者经面罩 5 L/min 吸入纯氧，麻醉诱导：舒芬太尼 0.3 μg/kg，丙泊酚 1.5~2 mg/kg，待睫毛反射消失、意识消失后静脉注射罗库溴铵 0.6 mg/kg，面罩辅助呼吸，可视喉镜下行气管插管术，连接呼吸机控制呼吸，吸入氧浓度 60%，容量控制模式，潮气量 6~8 mL/kg，频率 12~15 次/分，吸呼比 1：2，控制呼气末二氧化碳 PetCO2 在 35~40 mmHg。术中根据脑电监测的结果来调整麻醉深度，根据手术操作及患者的血流动力学反应追加镇痛药物，舒芬太尼 0.1~0.2 μg/kg 分次静推，瑞芬

太尼 0.05~0.2 μg/kg/min，七氟烷 1-3%吸入维持 BIS 值在 40~60 之间，罗库溴铵 0.1~0.2mg/kg 间段推注维持肌松。手术结束前 30 min 静推氟比洛芬酯 50mg，缝皮完成后停止七氟烷、瑞芬太尼使用。当 TOFc（四个成串刺激计数）≥ 3 时，根据患者分组给予新斯的明/格隆溴铵或舒更葡糖（具体见干预措施部分）。当患者的自我意识和神经反射恢复并达到拔管标准时，拔除气管插管，送至麻醉后恢复室（post anesthesia care unit, PACU）。麻醉护士对患者进行改良的 Aldrete 评分（包括：肌力、呼吸、循环、氧和、神志，每项 2 分，总分为 10 分），若 Aldrete 评分 ≥9 分，麻醉护士护送患者由 PACU 转至普通病房进行下一步治疗。

患者在诱导后给予地塞米松 5 mg，术毕给予帕洛诺司琼 0.075 mg 预防术后恶心呕吐。手术中给予氟比洛芬酯 50 mg，术后使用患者自控式静脉镇痛，镇痛输注泵内加入舒芬太尼 100 μg，使用 0.9%的生理盐水稀释至 100 mL，背景剂量速度设置为 1 mL/小时，自控剂量设置为 2 mL，锁时时间设置为 10 分钟。病区护士对手术患者进行每日数字疼痛量表（NRS）评分，若 NRS 评分 ≥4 分，则上报管床医生下达静滴氟比洛芬酯 50 mg 医嘱进行补救镇痛。

（1）术前量表评估

评估人员术前 1 天使用 MMSE 量表对计划行非心脏大手术的老年患者进行测试，该量表总分为 30 分，测试内容包括：时间和地点回忆、单词重复、算数（100 连续减 7）、言语表达、言语理解、简单指令运动[14]。认知障碍的判定标准为：文盲≤19，小学≤22，初中及以上≤26 [15]。

（2）术后量表评估

a.意识模糊评估法（3-minute Confusion Assessment Method, CAM）及严重性评估（CAM-S）：随访人员术后第 1 天至第 7 天或出院前对纳入的患者进行 POD 评估。CAM 的特征包括：①急性起病；②注意力不集中；③思维混乱；④意识改变。当特征①和特征②同时出现，且存在特征③或特征④时，临床诊断存在 POD[16]，CAM-S 进一步反映其严重程度[17, 18]。每天评估两次（08:00~10:00 区间和 19:00~21:00 区间各评估一次），包括周末和节假日。

b.认知功能电话问卷（10 items-Telephone Interview of Cognition Status，TICS-10）：电话随访人员在术后第 30 天对纳入的患者进行电话随访。TICS-10 对于神经认知功能延迟恢复评估有效，评估包括：时间定位（对日、月、年、星期和季节的认知）和数学计算（100 连续减去 7），总分为 10 分[19, 20]。

c.恢复质量量表（QoR-15）：该量表每项采用 0~10 分的计分方法，“0”提示一直都不能达到该种情况（状态不好），“10”提示一直都能达到该种情况（状态很好）。15 项相加得出总分，满分 150 分，总分越高提示术后恢复越好[21, 22]。

d.疼痛数字评估量表（NRS）：将一条直线平均分成 10 份，在每个点用数字 0～10 分表示疼痛依次加重的程度，0 分为无痛，10 分为剧痛。1~3 分提示轻度疼痛、4~6 分提示中度疼痛、7~10 分提示重度疼痛。

七、干预措施

当 TOFc（四个成串刺激计数）≥ 3 时，新斯的明组患者给予新斯的明 40 μg/kg+格隆溴铵 8 μg/kg。舒更葡糖组患者给予舒更葡糖 2 mg/kg。

观察指标：

1）术前：记录人口统计学特征（性别、年龄、身高、体重），教育水平、居住情况、术前基础疾病、MMSE 评分及术前检测结果。

2）术中：记录入室时患者的生命体征（HR、BP、SpO2），电脑自动采集术中生命体征数据；记录麻醉药物使用总量（丙泊酚、瑞芬太尼、舒芬太尼）、术中容量治疗、手术时间、拔管时间、PACU 停留时间。

3）术后：记录术后各量表评分、PCIA 内舒芬太尼使用量，记录术后住院天数，住院期间生存情况及并发症。

八、结局指标

1.主要结局指标为术后 7 天内或出院前 POD 的发生率。

2.次要结局：（1）POD 发作时间及占术后住院时间百分比；（2）CAM-S 评分峰值及总和 [23]；（3）术后 30 天 TICS-10 评分。

3.探索性结局：（1）疼痛：术后 24 及 48 小时静息和活动时的 NRS 评分；（2）镇痛：24 及 48 小时镇痛泵按压次数及补救镇痛次数；（3）恢复：术后 24 及 48 小时 QoR-15 评分；（4）术后 PONV；（5）非谵妄并发症（包括：低氧血症、肺水肿、肺部感染、呼吸衰竭、心肌梗死、新发房颤、心力衰竭、胃肠出血、脑卒中、肾功能衰竭、失血性休克、败血症、感染性休克、吻合口瘘、二次手术）；（6）术后住院时间；（7）30 天死亡率。

九、随访计划

分别于术后 1 天至 7 天或出院前进行病房随访，30 天进行电话随访。随访内容为 CAM 及 CAM-S 评分、QoR-15 评分、TICS-10 评分、术后并发症，生存情况。

十、流程图

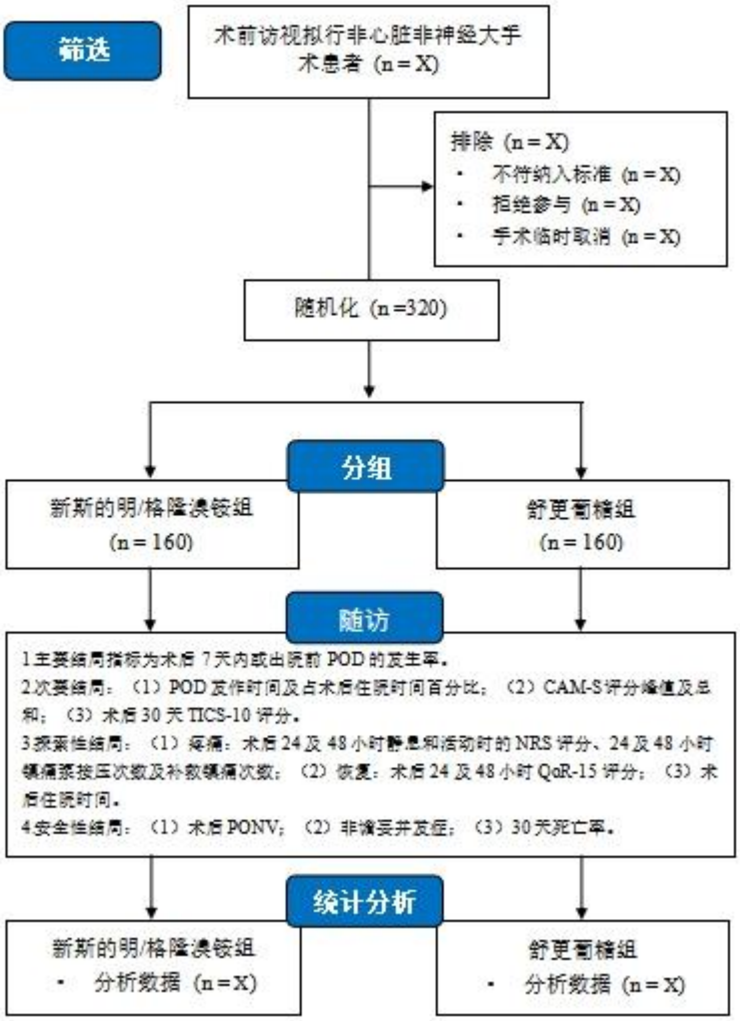

十一、研究的安全性监测、不良事件的处理方案

本研究中的干预措施以及其他的麻醉用药，均是临床上常规使用的麻醉药物、麻醉方法，将不会给受试者增加超出常规诊疗以外的风险。术中由上级主治及以上级别麻醉医生对整个麻醉过程进行安全性监测。可能存在的不良反应和治疗措施如下：高血压（MAP 升高幅度超过基础值 30%）和心动过速（HR>100 次/min），在麻醉深度足够的情况下静脉注射佩尔地平 0.5mg/次或艾司洛尔 20mg/次；低血压（MAP 降低幅度超过基础值 30%），静脉输注液体，注射麻黄碱 6mg/次或者去氧肾上腺素 50µg/次；心动过缓（HR＜45 次/min），静脉注射格隆溴铵 0.2mg/次。术后疼痛补救治疗方案：如患者 VAS 评分大于等于 4 分，则给予舒芬太尼 5µg 静脉滴注，必要时 24 小时内可重复一次。术后恶心呕吐补救治疗方案：患者术后如发生恶心呕吐，可根据情况给予帕洛诺司琼、胃复安等治疗。记录任何不良事件，包括类型、时间、持续时间、处理方式；持续随访直到完全解决或治疗终止。如果出现任何严重不良事件，将停止研究方案并立即开始治疗；如果主治麻醉医师或主要研究者认为必要，可暂时或永久停

止研究方案，研究中断的时间和原因将记录在病例记录表（CRF）中；如有任何严重不良事件，除上述积极治疗和记录外，在 24 小时内以书面报告通知主要研究者和伦理委员会。

十二、研究数据的管理与统计分析

所有数据由对研究分组不知情的独立研究人员记录在病例报告表（CRF）之中，然后上传到电子数据库。研究负责人将确保数据的准确性和完整性，并且采集的数据受到监管。统计学分析采用 SPSS, version 25.0 (IBM SPSS)。统计人员采用 SPSS 25 进行数据间的统计分析。连续变量统计：以均数±标准差或中位数（四分位间距）显示，组间比较的检验方法根据数据分布确定（独立样本 t 检验、Wilcoxon 秩和检验或 Kruskal-Wallis 非参数检验）。分类变量统计：以数字（百分比）显示，组间比较使用 χ2 检验或 Fisher 精确检验。采用 logistic 回归模型评估与 POD 相关的潜在危险因素。生存分析：使用 Cox 检验和 Kaplan-Meier 生存曲线对 POD 的发生时间进行分析，组间比较的效应量为风险比（hazard ratio, HR）和 95%CI。双侧 $p<0.05$ 被认为具有统计学意义。

十二、参考文献

[1] Evered L, Silbert B, Knopman DS, et al. Recommendations for the Nomenclature of Cognitive Change Associated with Anaesthesia and Surgery-2018[J]. Anesthesiology, 2018, 129(5): 872-879.DOI: 10.1097/aln.0000000000002334

[2] Ho MH, Nealon J, Igwe E, et al. Postoperative Delirium in Older Patients: A Systematic Review of Assessment and Incidence of Postoperative Delirium[J]. Worldviews Evid Based Nurs, 2021, 18(5): 290-301.DOI: 10.1111/wvn.12536

[3] Swarbrick CJ, Partridge JSL. Evidence-based strategies to reduce the incidence of postoperative delirium: a narrative review[J]. Anaesthesia, 2022, 77 Suppl 1: 92-101.DOI: 10.1111/anae.15607

[4] Moller JT, Cluitmans P, Rasmussen LS, et al. Long-term postoperative cognitive dysfunction in the elderly ISPOCD1 study. ISPOCD investigators. International Study of Post-Operative Cognitive Dysfunction[J]. Lancet, 1998, 351(9106): 857-861.DOI: 10.1016/s0140-6736(97)07382-0

[5] Li T, Li J, Yuan L, et al. Effect of Regional vs General Anesthesia on Incidence of Postoperative Delirium in Older Patients Undergoing Hip Fracture Surgery: The RAGA Randomized Trial[J]. Jama, 2022, 327(1): 50-58.DOI: 10.1001/jama.2021.22647

[6] Ploeger BA, Smeets J, Strougo A, Drenth HJ, Ruigt G, Houwing N, Danhof M. Pharmacokinetic-pharmacodynamic model for the reversal of neuromuscular blockade by sugammadex. Anesthesiology. 2009 Jan;110(1):95-105. DOI: 10.1097/ALN.0b013e318190bc32.

[7] Aniskevich S, Leone BJ, Brull SJ. Sugammadex: a novel approach to reversal of neuromuscular blockade. Expert Rev Neurother. 2011;11(2):185-98. DOI: 10.1586/ern.11.2.

[8] Deng C, Yang L, Sun D, Feng Y, Sun Z, Li J. Influence of Neostigmine on Early Postoperative Cognitive Dysfunction in Older Adult Patients Undergoing Noncardiac Surgery: A Double-Blind, Placebo-Controlled, Randomized Controlled Trial. Anesth Analg. 2024 Mar 1;138(3):589-597. DOI:

10.1213/ANE.0000000000006687.

[9] Zhu B, Sun D, Yang L, Sun Z, Feng Y, Deng C. The effects of neostigmine on postoperative cognitive function and inflammatory factors in elderly patients - a randomized trial. BMC Geriatr. 2020 Oct 6;20(1):387. DOI: 10.1186/s12877-020-01793-4.

[10] Maldonado JR. Delirium pathophysiology: An updated hypothesis of the etiology of acute brain failure. Int J Geriatr Psychiatry. 2018 Nov;33(11):1428-1457. DOI: 10.1002/gps.4823.

[11] Adam EH, Haas V, Lindau S, Zacharowski K, Scheller B. Cholinesterase alterations in delirium after cardiosurgery: a German monocentric prospective study. BMJ Open. 2020;10(1):e031212. DOI: 10.1136/bmjopen-2019-031212.

[12] Bash LD, Turzhitsky V, Mark RJ, et al. Post-operative urinary retention is impacted by neuromuscular block reversal agent choice: A retrospective cohort study in US hospital setting. J Clin Anesth. 2024; 93:111344. DOI: 10.1016/j.jclinane.2023.111344.

[13] Simpson KH, Smith RJ, Davies LF. Comparison of the effects of atropine and glycopyrrolate on cognitive function following general anaesthesia. Br J Anaesth. 1987 Aug;59(8):966-9. DOI: 10.1093/bja/59.8.966.

[14] Jia X, Wang Z, Huang F, et al. A comparison of the Mini-Mental State Examination (MMSE) with the Montreal Cognitive Assessment (MoCA) for mild cognitive impairment screening in Chinese middle-aged and older population: a cross-sectional study[J]. BMC Psychiatry, 2021, 21(1): 485.DOI: 10.1186/s12888-021-03495-6

[15] Lu J, Li D, Li F, et al. Montreal cognitive assessment in detecting cognitive impairment in Chinese elderly individuals: a population-based study. J Geriatr Psychiatry Neurol. 2011:184-90. DOI: 10.1177/0891988711422528.

[16] Marcantonio ER, Ngo LH, O'Connor M, et al. 3D-CAM: derivation and validation of a 3-minute diagnostic interview for CAM-defined delirium: a cross-sectional diagnostic test study[J]. Ann Intern Med, 2014, 161(8): 554-561.DOI: 10.7326/m14-0865

[17] Inouye SK, Kosar CM, Tommet D, et al. The CAM-S: development and validation of a new scoring system for delirium severity in 2 cohorts. Ann Intern Med. 2014, 160(8):526-533. DOI: 10.7326/M13-1927.

[18] Mei X, Chen Y, Zheng H, Shi Z, et al. The Reliability and Validity of the Chinese Version of Confusion Assessment Method Based Scoring System for Delirium Severity (CAM-S). J Alzheimers Dis. 2019;69(3):709-716. DOI: 10.3233/JAD-181288.

[19] Li J, Cacchione PZ, Hodgson N, et al. Afternoon Napping and Cognition in Chinese Older Adults: Findings from the China Health and Retirement Longitudinal Study Baseline Assessment[J]. J Am Geriatr Soc, 2017, 65(2): 373-380.DOI: 10.1111/jgs.14368

[20] Hua J, Dong J, Chen GC, et al. Trends in cognitive function before and after stroke in China. BMC Med. 2023, 21(1):204. DOI: 10.1186/s12916-023-02908-5.

[21] Wessels E, Perrie H, Scribante J, et al. Quality of recovery in the perioperative setting: A narrative review[J]. J Clin Anesth, 2022, 78: 110685.DOI: 10.1016/j.jclinane.2022.110685

[22] Kleif J, Gögenur I. Severity classification of the quality of recovery-15 score-An observational

study[J]. J Surg Res, 2018, 225: 101-107.DOI: 10.1016/j.jss.2017.12.040

[23] Vasunilashorn SM, Marcantonio ER, Gou Y, et al. Quantifying the Severity of a Delirium Episode Throughout Hospitalization: the Combined Importance of Intensity and Duration. J Gen Intern Med. 2016 ;31(10):1164-71. DOI: 10.1007/s11606-016-3671-9.
