# Supplementary material for: Effect of neostigmine/glycopyrrolate versus sugammadex on postoperative delirium in older adults: A triple-masked, randomized, controlled trial protocol
Source: PLoS One. 2026 Apr 1;21(4):e0346523. doi: 10.1371/journal.pone.0346523 (PMC13042718; doi:10.1371/journal.pone.0346523)
Supplement: S2 File — (DOCX) [file pone.0346523.s002.docx]

**Research Protocol**

Version Number: 1.0

Version Date: October 25, 2024

**I. Research Project Title**

Effect of Neostigmine/Glycopyrrolate versus Sugammadex on Postoperative Delirium in Elderly Patients Undergoing Non-Cardiac Major Surgery: A Randomized Controlled Study

**II. Research Background**

Postoperative delirium (POD) is a clinical acute brain syndrome characterized by decreased attention, fluctuating levels of consciousness, and cognitive dysfunction, and is one of the most common postoperative complications in the elderly. According to the revised concept of postoperative cognitive dysfunction in 2018, POD refers to acute cognitive impairment occurring within 1 week after surgery[1]. The prevalence of POD varies after surgeries of different risks, and in elderly patients undergoing non-cardiac major surgery, the prevalence of POD is 20%~30%[2]. POD brings many obstacles to patients' postoperative recovery, such as prolonged hospital stay, increased risk of other complications, increased medical costs, and increased mortality. The main causes of POD include central nervous system damage (hypoxia, hypoglycemia, metabolic disorders, etc.) and abnormal stress responses (systemic inflammatory response, etc.), and these two mechanisms have more obvious effects in the brains of the elderly[3], leading to a higher prevalence of postoperative POD in the elderly. The main risk factors for POD include advanced age, prolonged anesthesia duration, low educational level, reoperation, infection, and respiratory complications[4].

Anesthetic drugs are also associated with the occurrence of POD[5], and muscle relaxant antagonists are one of them. Currently, the commonly used muscle relaxant antagonists are sugammadex and neostigmine, which have different mechanisms of action. Sugammadex is a modified γ-cyclodextrin that forms a complex with the neuromuscular blockers rocuronium or vecuronium, reducing the number of neuromuscular blockers bound to nicotinic receptors at the neuromuscular junction, thereby reversing rocuronium or vecuronium-induced neuromuscular block. The drug itself does not affect the function of acetylcholine at the neuromuscular junction[6, 7]. As a cholinesterase inhibitor, neostigmine increases the concentration of acetylcholine and exerts an antagonistic effect on the residual muscle relaxation caused by non-depolarizing muscle relaxants. Increasing the acetylcholine content in the synaptic cleft by cholinesterase inhibitors is also the main mechanism for improving dementia and cognitive function, and such drugs (such as donepezil and rivastigmine) are currently the first-line treatment drugs for mild to moderate Alzheimer's disease. Regarding neostigmine, current studies have shown that this drug may improve postoperative cognition[8-11]. At the same time, to counteract the muscarinic effects caused by neostigmine, anticholinergic drugs are usually used in combination. Among them, glycopyrrolate is a quaternary ammonium anticholinergic drug that is not easily permeable to lipid membranes such as the blood-brain barrier. Compared with tertiary ammonium anticholinergic drugs atropine and scopolamine hydrobromide, glycopyrrolate has fewer central nervous system-related adverse reactions[12, 13]. However, current research on the effects of neostigmine/glycopyrrolate and sugammadex in antagonizing muscle relaxation after general anesthesia on postoperative cognitive function in elderly patients is still limited.

Therefore, this study intends to carry out a prospective randomized controlled clinical study. By using neostigmine/glycopyrrolate or sugammadex to antagonize neuromuscular block after surgery, the effects on the incidence (primary outcome), severity, duration, postoperative pain, etc. (secondary outcomes) of postoperative POD in elderly patients undergoing non-cardiac major surgery will be evaluated, so as to provide a basis for perioperative anesthetic medication management in elderly patients undergoing non-cardiac major surgery and promote the improvement of perioperative recovery quality.

**III. Research Objectives**

To evaluate whether neostigmine/glycopyrrolate antagonism of neuromuscular block can reduce the incidence of postoperative delirium in elderly patients undergoing non-cardiac major surgery, thereby providing a better anesthetic medication strategy for improving patient prognosis and enhancing patient rehabilitation quality.

**IV. Research Design** (Including Overall Research Design, Sample Size, Number of Participating Institutions, Research Steps, Research Timeframe, etc.)

**1. Overall Design**

This study is an investigator-initiated, single-center, prospective, randomized controlled clinical study.

**2. Sample Size Calculation**

The incidence of postoperative delirium in elderly patients undergoing non-cardiac major surgery is about 20%~30%. We assume that the incidence of POD in the sugammadex group is 25%, and combined with the latest research, neostigmine can improve postoperative cognitive function by 50%. PASS 15 software (NCSS, LLC. Kaysville, Utah, USA) was used for sample size calculation. A two-tailed test with α=0.05 and a power of 80% was adopted. A total of 304 samples are needed. Considering a 5% loss to follow-up rate, 320 patients are planned to be included, with 160 patients in each group.


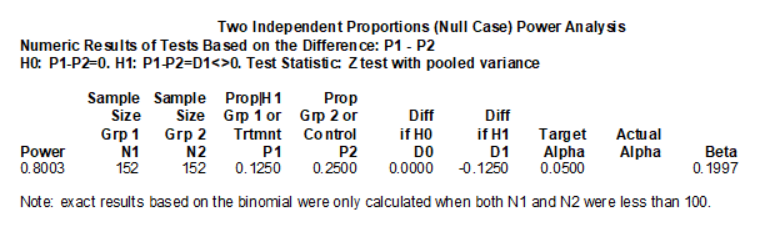


**3. Participating Institution**

The First Affiliated Hospital of Soochow University

**4. Ethics and Registration**

After obtaining ethical approval, online registration will be conducted at the Chinese Clinical Trial Registry, a WHO Level 1 registration institution, to obtain a clinical trial registration number. Clinical trial registration will be completed before enrolling the first patient. All enrolled patients will be fully informed of the research process and sign a written informed consent form.

**5. Research Steps and Timeframe**

After ethical registration, preliminary screening of patients will be carried out according to the study plan, informed consent forms will be signed, patients will be enrolled according to a computer-generated random list, anesthesia and surgery will be performed, and follow-up visits will be conducted at specified time points after surgery until the patient is discharged from the hospital. The study is expected to be completed within 1.5 years.

**V. Research Population** (Including Inclusion Criteria, Exclusion Criteria, Withdrawal Criteria, Termination Criteria, etc.)

**1. Inclusion Criteria**

1) Age ≥ 65 years old, regardless of gender;

2) American Society of Anesthesiologists (ASA) classification Grade I-III;

3) Scheduled for elective non-cardiac and non-neurological major surgery (thoracic surgery, major abdominal surgery, urological surgery, extremity and joint surgery, and spinal surgery), with an expected operation time ≥ 1.5 hours;

4) Planned to have tracheal intubation extubated after surgery and transferred to the surgical ward, with an expected postoperative hospital stay ≥ 2 days;

5) Clearly understand the research process, voluntarily participate, and sign the informed consent form.

**2. Exclusion Criteria**

1) Allergy to the drugs used in the study;

2) Presence of contraindications to neostigmine (epilepsy, intestinal obstruction, urinary tract obstruction, bronchial asthma, glaucoma), paralysis, or neuromuscular diseases;

3) Severe liver dysfunction (Child-Pugh Class C), renal failure;

4) Long-term use of cholinesterase inhibitors, anticholinergic drugs, or psychotropic drugs;

5) Inability to communicate effectively or refusal to participate in the study.

**3. Withdrawal or Termination Criteria**

1) Severe adverse reactions during surgery (such as life-threatening massive hemorrhage, anaphylactic shock, etc.), reoperation; 2) Transfer to the intensive care unit with a tube after surgery; 3) The subject or agent withdraws the informed consent form.

**VI. Research Implementation Intervention Plan**

Patients will be visited one day before surgery to sign the informed consent form for anesthesia and research projects, explain the use of relevant rating scales to the patients, collect basic data (age, gender, educational level, BMI, comorbidities), and conduct Mini-Mental State Examination (MMSE) scoring. No preoperative medication will be used on the day of surgery, and preoperative fasting will be for 6 hours. Standard monitoring of patients after entering the operating room includes: electrocardiogram (ECG), pulse oximetry (SpO2), non-invasive blood pressure (NIBP), BIS monitoring for anesthetic depth, and muscle relaxation monitoring. Peripheral venous catheterization will be completed, and basic fluid infusion (balanced solution, 5 mL/kg/h) will be started. Patients will inhale pure oxygen at 5 L/min through a mask. Anesthesia induction: sufentanil 0.3 μg/kg, propofol 1.5~2 mg/kg. After the disappearance of eyelash reflex and consciousness, rocuronium 0.6 mg/kg will be injected intravenously, mask-assisted ventilation will be performed, tracheal intubation will be performed under video laryngoscopy, and the ventilator will be connected for controlled ventilation. The inspired oxygen concentration is 60%, volume-controlled mode, tidal volume 6~8 mL/kg, frequency 12~15 times/min, inspiratory-expiratory ratio 1:2, and end-tidal carbon dioxide (PetCO2) will be controlled at 35~40 mmHg. During the operation, the depth of anesthesia will be adjusted according to the results of electroencephalographic monitoring, and analgesic drugs will be added according to the surgical operation and the patient's hemodynamic response. Sufentanil 0.1~0.2 μg/kg will be injected intravenously in divided doses, remifentanil 0.05~0.2 μg/kg/min, sevoflurane 1-3% inhalation to maintain BIS value between 40~60, and rocuronium 0.1~0.2 mg/kg will be injected intermittently to maintain muscle relaxation. Flurbiprofen axetil 50 mg will be injected intravenously 30 minutes before the end of surgery, and sevoflurane and remifentanil will be stopped after suture completion. When TOFc (train-of-four count) ≥ 3, neostigmine/glycopyrrolate or sugammadex will be administered according to the patient's group (see the intervention measures section for details). When the patient's self-consciousness and neurological reflexes recover and meet the extubation criteria, the tracheal tube will be extubated and the patient will be sent to the post-anesthesia care unit (PACU). Anesthesia nurses will perform a modified Aldrete score on the patient (including: muscle strength, respiration, circulation, oxygenation, consciousness, 2 points for each item, total score 10 points). If the Aldrete score ≥ 9 points, the anesthesia nurse will escort the patient from the PACU to the general ward for further treatment.

Patients will be given dexamethasone 5 mg after induction, and palonosetron 0.075 mg at the end of surgery to prevent postoperative nausea and vomiting. Flurbiprofen axetil 50 mg will be given during the operation, and patient-controlled intravenous analgesia (PCIA) will be used after surgery. The analgesic infusion pump will contain sufentanil 100 μg, diluted to 100 mL with 0.9% normal saline. The background infusion rate is set at 1 mL/hour, the patient-controlled dose is set at 2 mL, and the lockout time is set at 10 minutes. Ward nurses will perform daily Numerical Rating Scale (NRS) scoring for surgical patients. If the NRS score ≥ 4 points, the attending physician will be reported to issue a medical order for intravenous infusion of flurbiprofen axetil 50 mg for rescue analgesia.

(1) Preoperative Scale Assessment

Assessors will use the MMSE scale to test elderly patients scheduled for non-cardiac major surgery 1 day before surgery. The total score of this scale is 30 points. The test content includes: time and place recall, word repetition, arithmetic (subtracting 7 consecutively from 100), verbal expression, verbal comprehension, and simple command movement[14]. The criteria for judging cognitive impairment are: illiterate ≤ 19, primary school ≤ 22, junior high school and above ≤ 26[15].

(2) Postoperative Scale Assessment

a. Confusion Assessment Method (CAM) and Severity Assessment (CAM-S): Follow-up personnel will assess POD in enrolled patients from postoperative day 1 to day 7 or before discharge. The characteristics of CAM include: ① acute onset; ② inattention; ③ disorganized thinking; ④ altered consciousness. When both characteristic ① and characteristic ② appear, and characteristic ③ or ④ exists, POD is clinically diagnosed[16], and CAM-S further reflects its severity[17, 18]. Assessments will be performed twice a day (once between 08:00~10:00 and once between 19:00~21:00), including weekends and holidays.

b. 10-item Telephone Interview of Cognition Status (TICS-10): Telephone follow-up personnel will conduct telephone follow-up on enrolled patients on postoperative day 30. TICS-10 is effective for assessing delayed recovery of neurocognitive function. The assessment includes: time orientation (cognition of day, month, year, day of the week, and season) and mathematical calculation (subtracting 7 consecutively from 100), with a total score of 10 points[19, 20].

c. Quality of Recovery-15 (QoR-15) Scale: Each item of this scale is scored on a 0~10 scale, where "0" indicates that this situation can never be achieved (poor state), and "10" indicates that this situation can always be achieved (good state). The total score is obtained by adding 15 items, with a maximum score of 150 points. A higher total score indicates better postoperative recovery[21, 22].

d. Numerical Rating Scale (NRS) for Pain: A straight line is evenly divided into 10 parts, and each point is marked with numbers 0~10 to indicate the increasing degree of pain. 0 points means no pain, and 10 points means severe pain. 1~3 points indicate mild pain, 4~6 points indicate moderate pain, and 7~10 points indicate severe pain.

**VII. Intervention Measures**

When TOFc (train-of-four count) ≥ 3, patients in the neostigmine group will receive neostigmine 40 μg/kg + glycopyrrolate 8 μg/kg. Patients in the sugammadex group will receive sugammadex 2 mg/kg.

1) Preoperative: Record demographic characteristics (gender, age, height, weight), educational level, living conditions, preoperative underlying diseases, MMSE score, and preoperative test results.

2) Intraoperative: Record the patient's vital signs (HR, BP, SpO2) upon entering the operating room, and the computer will automatically collect intraoperative vital sign data; record the total dosage of anesthetic drugs (propofol, remifentanil, sufentanil), intraoperative volume therapy, operation time, extubation time, and PACU stay time.

3) Postoperative: Record postoperative scores of various scales, the dosage of sufentanil in PCIA, postoperative hospital stay, survival status during hospitalization, and complications.

**VIII. Outcome measurements**

1. The primary outcome indicator is the incidence of POD within 7 days after surgery or before discharge.

2. Secondary outcomes: (1) Onset time of POD and percentage of postoperative hospital stay; (2) Peak value and sum of CAM-S scores[23]; (3) TICS-10 score on postoperative day 30.

3. Exploratory outcomes: (1) Pain: NRS scores at rest and during activity at 24 and 48 hours after surgery; (2) Analgesia: Number of analgesic pump presses and rescue analgesia times at 24 and 48 hours; (3) Recovery: QoR-15 scores at 24 and 48 hours after surgery; (4) Postoperative PONV; (5) Non-delirium complications (including: hypoxemia, pulmonary edema, pulmonary infection, respiratory failure, myocardial infarction, new-onset atrial fibrillation, heart failure, gastrointestinal bleeding, stroke, renal failure, hemorrhagic shock, sepsis, septic shock, anastomotic leakage, reoperation); (6) Postoperative hospital stay; (7) 30-day mortality.

**IX. Follow-up Plan**

Ward follow-up will be conducted from postoperative day 1 to day 7 or before discharge, and telephone follow-up will be conducted on postoperative day 30. The follow-up content includes CAM and CAM-S scores, QoR-15 scores, TICS-10 scores, postoperative complications, and survival status.

**X. Flow Chart**

Screening: Preoperative visit to patients scheduled for non-cardiac and non-neurological major surgery (n=X)

Exclusion (n=X): Failure to meet inclusion criteria (n=X), Refusal to participate (n=X), Temporary cancellation of surgery (n=X)

Randomization (n=320)

Grouping: Neostigmine/Glycopyrrolate Group (n=160), Sugammadex Group (n=160)

Follow-up: 1. Primary outcome: Incidence of POD within 7 days after surgery or before discharge. 2. Secondary outcomes: (1) Onset time of POD and percentage of postoperative hospital stay; (2) Peak value and sum of CAM-S scores; (3) TICS-10 score on postoperative day 30. 3. Exploratory outcomes: (1) Pain: NRS scores at rest and during activity at 24 and 48 hours after surgery; number of analgesic pump presses and rescue analgesia times at 24 and 48 hours; (2) Recovery: QoR-15 scores at 24 and 48 hours after surgery; (3) Safety: (1) Postoperative PONV; (2) Non-delirium complications; (3) 30-day mortality; (4) Postoperative hospital stay.

Statistical Analysis: Neostigmine/Glycopyrrolate Group (Analyzed data n=X), Sugammadex Group (Analyzed data n=X)

**XI. Research Safety Monitoring and Adverse Event Handling Plan**

The intervention measures and other anesthetic drugs used in this study are clinically routine anesthetic drugs and anesthesia methods, and will not increase risks beyond routine diagnosis and treatment for the subjects. During the operation, anesthesiologists at or above the attending physician level will monitor the safety of the entire anesthesia process. Possible adverse reactions and treatment measures are as follows: Hypertension (MAP increase exceeding 30% of the baseline value) and tachycardia (HR>100 beats/min): Under sufficient depth of anesthesia, inject perindopril 0.5 mg/time or esmolol 20 mg/time intravenously; Hypotension (MAP decrease exceeding 30% of the baseline value): Intravenous fluid infusion, inject ephedrine 6 mg/time or phenylephrine 50 μg/time; Bradycardia (HR<45 beats/min): Inject glycopyrrolate 0.2 mg/time intravenously. Postoperative pain rescue treatment plan: If the patient's VAS score is ≥ 4 points, 5 μg of sufentanil will be given intravenously, which can be repeated once within 24 hours if necessary. Postoperative nausea and vomiting rescue treatment plan: If the patient experiences nausea and vomiting after surgery, palonosetron, metoclopramide, etc. can be given according to the situation. Record any adverse events, including type, time, duration, and treatment method; continuous follow-up until complete resolution or termination of treatment. If any serious adverse event occurs, the research protocol will be stopped and treatment will be started immediately; if the attending anesthesiologist or principal investigator deems it necessary, the research protocol can be temporarily or permanently stopped.

The time and reason for the research interruption will be recorded in the Case Report Form (CRF); in case of any serious adverse event, in addition to the above active treatment and recording, a written report will be submitted to the principal investigator and the ethics committee within 24 hours.

**XII. Research Data Management and Statistical Analysis**

All data will be recorded in the Case Report Form (CRF) by independent researchers who are unaware of the research grouping, and then uploaded to an electronic database. The research director will ensure the accuracy and completeness of the data, and the collected data will be supervised. Statistical analysis will be performed using SPSS, version 25.0 (IBM SPSS). Statisticians will use SPSS 25 for statistical analysis of the data. Continuous variables: Expressed as mean ± standard deviation or median (interquartile range), and the test method for intergroup comparison will be determined according to the data distribution (independent sample t-test, Wilcoxon rank-sum test, or Kruskal-Wallis nonparametric test). Categorical variables: Expressed as numbers (percentages), and χ2 test or Fisher's exact test will be used for intergroup comparison. Logistic regression model will be used to evaluate potential risk factors associated with POD. Survival analysis: Cox test and Kaplan-Meier survival curve will be used to analyze the occurrence time of POD, and the effect size for intergroup comparison is hazard ratio (HR) and 95% CI. A two-tailed p<0.05 is considered statistically significant.

**XIII. References**

[1] Evered L, Silbert B, Knopman DS, et al. Recommendations for the Nomenclature of Cognitive Change Associated with Anaesthesia and Surgery-2018[J]. Anesthesiology, 2018, 129(5): 872-879. DOI: 10.1097/aln.0000000000002334

[2] Ho MH, Nealon J, Igwe E, et al. Postoperative Delirium in Older Patients: A Systematic Review of Assessment and Incidence of Postoperative Delirium[J]. Worldviews Evid Based Nurs, 2021, 18(5): 290-301. DOI: 10.1111/wvn.12536

[3] Swarbrick CJ, Partridge JSL. Evidence-based strategies to reduce the incidence of postoperative delirium: a narrative review[J]. Anaesthesia, 2022, 77 Suppl 1: 92-101. DOI: 10.1111/anae.15607

[4] Moller JT, Cluitmans P, Rasmussen LS, et al. Long-term postoperative cognitive dysfunction in the elderly ISPOCD1 study. ISPOCD investigators. International Study of Post-Operative Cognitive Dysfunction[J]. Lancet, 1998, 351(9106): 857-861. DOI: 10.1016/s0140-6736(97)07382-0

[5] Li T, Li J, Yuan L, et al. Effect of Regional vs General Anesthesia on Incidence of Postoperative Delirium in Older Patients Undergoing Hip Fracture Surgery: The RAGA Randomized Trial[J]. Jama, 2022, 327(1): 50-58. DOI: 10.1001/jama.2021.22647

[6] Ploeger BA, Smeets J, Strougo A, Drenth HJ, Ruigt G, Houwing N, Danhof M. Pharmacokinetic-pharmacodynamic model for the reversal of neuromuscular blockade by sugammadex. Anesthesiology. 2009 Jan;110(1):95-105. DOI: 10.1097/ALN.0b013e318190bc32.

[7] Aniskevich S, Leone BJ, Brull SJ. Sugammadex: a novel approach to reversal of neuromuscular blockade. Expert Rev Neurother. 2011;11(2):185-98. DOI: 10.1586/ern.11.2.

[8] Deng C, Yang L, Sun D, Feng Y, Sun Z, Li J. Influence of Neostigmine on Early Postoperative Cognitive Dysfunction in Older Adult Patients Undergoing Noncardiac Surgery: A Double-Blind, Placebo-Controlled, Randomized Controlled Trial. Anesth Analg. 2024 Mar 1;138(3):589-597. DOI: 10.1213/ANE.0000000000006687.

[9] Zhu B, Sun D, Yang L, Sun Z, Feng Y, Deng C. The effects of neostigmine on postoperative cognitive function and inflammatory factors in elderly patients - a randomized trial. BMC Geriatr. 2020 Oct 6;20(1):387. DOI: 10.1186/s12877-020-01793-4.

[10] Maldonado JR. Delirium pathophysiology: An updated hypothesis of the etiology of acute brain failure. Int J Geriatr Psychiatry. 2018 Nov;33(11):1428-1457. DOI: 10.1002/gps.4823.

[11] Adam EH, Haas V, Lindau S, Zacharowski K, Scheller B. Cholinesterase alterations in delirium after cardiosurgery: a German monocentric prospective study. BMJ Open. 2020;10(1):e031212. DOI: 10.1136/bmjopen-2019-031212.

[12] Bash LD, Turzhitsky V, Mark RJ, et al. Post-operative urinary retention is impacted by neuromuscular block reversal agent choice: A retrospective cohort study in US hospital setting. J Clin Anesth. 2024; 93:111344. DOI: 10.1016/j.jclinane.2023.111344.

[13] Simpson KH, Smith RJ, Davies LF. Comparison of the effects of atropine and glycopyrrolate on cognitive function following general anaesthesia. Br J Anaesth. 1987 Aug;59(8):966-9. DOI: 10.1093/bja/59.8.966.

[14] Jia X, Wang Z, Huang F, et al. A comparison of the Mini-Mental State Examination (MMSE) with the Montreal Cognitive Assessment (MoCA) for mild cognitive impairment screening in Chinese middle-aged and older population: a cross-sectional study[J]. BMC Psychiatry, 2021, 21(1): 485. DOI: 10.1186/s12888-021-03495-6

[15] Lu J, Li D, Li F, et al. Montreal cognitive assessment in detecting cognitive impairment in Chinese elderly individuals: a population-based study. J Geriatr Psychiatry Neurol. 2011:184-90. DOI: 10.1177/0891988711422528.

[16] Marcantonio ER, Ngo LH, O'Connor M, et al. 3D-CAM: derivation and validation of a 3-minute diagnostic interview for CAM-defined delirium: a cross-sectional diagnostic test study[J]. Ann Intern Med, 2014, 161(8): 554-561. DOI: 10.7326/m14-0865

[17] Inouye SK, Kosar CM, Tommet D, et al. The CAM-S: development and validation of a new scoring system for delirium severity in 2 cohorts. Ann Intern Med. 2014, 160(8):526-533. DOI: 10.7326/M13-1927.

[18] Mei X, Chen Y, Zheng H, Shi Z, et al. The Reliability and Validity of the Chinese Version of Confusion Assessment Method Based Scoring System for Delirium Severity (CAM-S). J Alzheimers Dis. 2019;69(3):709-716. DOI: 10.3233/JAD-181288.

[19] Li J, Cacchione PZ, Hodgson N, et al. Afternoon Napping and Cognition in Chinese Older Adults: Findings from the China Health and Retirement Longitudinal Study Baseline Assessment[J]. J Am Geriatr Soc, 2017, 65(2): 373-380. DOI: 10.1111/jgs.14368

[20] Hua J, Dong J, Chen GC, et al. Trends in cognitive function before and after stroke in China. BMC Med. 2023, 21(1):204. DOI: 10.1186/s12916-023-02908-5.

[21] Wessels E, Perrie H, Scribante J, et al. Quality of recovery in the perioperative setting: A narrative review[J]. J Clin Anesth, 2022, 78: 110685. DOI: 10.1016/j.jclinane.2022.110685

[22] Kleif J, Gögenur I. Severity classification of the quality of recovery-15 score-An observational study[J]. J Surg Res, 2018, 225: 101-107. DOI: 10.1016/j.jss.2017.12.040

[23] Vasunilashorn SM, Marcantonio ER, Gou Y, et al. Quantifying the Severity of a Delirium Episode Throughout Hospitalization: the Combined Importance of Intensity and Duration. J Gen Intern Med. 2016 ;31(10):1164-71. DOI: 10.1007/s11606-016-3671-9.
