# Supplementary material for: Effect of neostigmine/glycopyrrolate versus sugammadex on postoperative delirium in older adults: A triple-masked, randomized, controlled trial protocol
Source: PLoS One. 2026 Apr 1;21(4):e0346523. doi: 10.1371/journal.pone.0346523 (PMC13042718; doi:10.1371/journal.pone.0346523)
Supplement: Attachment — Submitted filename: Response to Reviewers.docx [file pone.0346523.s005.docx]

**Additional Editor Comments:**

This protocol describes a well-designed, triple-masked, randomized trial with a clearly defined primary endpoint. Key methodological concerns requiring clarification before implementation include: the handling of missing outcome data, which currently aligns with a modified intention-to-treat approach and risks bias if missing non-randomly;

Response: We appreciate this important suggestion. Our recently completed pilot trial (Long et al., Anesth Analg 2024, PMID: 37874773) employed an identical assessment protocol (twice-daily 3D-CAM evaluations by the same trained research team at our institution) in a comparable elderly surgical population (major non-cardiac surgery, mean age 69.6 years). In that study, POD assessments were completed without exception in all 78 randomized patients (100% ascertainment). Based on this, we expect minimal missing data (< 5%) in the current trial and have retained the complete case analysis as primary. Comprehensive sensitivity analyses will also be conducted to address any unforeseen missingness.

We have clarified this in the Statistical Analysis: “The primary analysis will be conducted in a modified intention-to-treat population, defined as all randomized patients who undergo surgery and have at least one postoperative POD assessment. In our recently completed pilot trial using identical assessment protocols, twice-daily 3D-CAM evaluations were completed without exception in all 78 older surgical patients (100% ascertainment). Based on this empirical evidence, we expect minimal missing data (< 5%) and will perform the primary analysis on a complete case basis. To assess robustness to any unforeseen missingness, we will conduct: (1) worst-case scenario analysis (all missing data assumed to be POD in the neostigmine/glycopyrrolate group and no POD in the sugammadex group); (2) best-case scenario analysis (reverse of worst-case); and (3) per-protocol analysis (excluding patients with major protocol deviations). If the observed missing data rate exceeds 10%, an additional sensitivity analysis using multiple imputation will be conducted.” *(see Methods, Statistical analysis, lines 218‒230)*

the detailed statistical specification for the time-to-event analysis of POD, including the handling of interval-censored data and discharges;

Response: Thank you. We have clarified the Kaplan-Meier method to analyze time to POD, including the survival model, time origin, and assumptions: “Kaplan–Meier curves will be used to describe time to first POD, with the day of surgery set as day 0. Time-to-event analysis will be conducted on a discrete-time scale (calendar days). The treatment effect will be estimated with a Cox proportional-hazards model. As a sensitivity analysis for competing risks (death and early discharge), we will estimate cumulative incidence functions using the Fine-Gray model. If the proportional hazards assumption is violated (Schoenfeld residuals test *P* < 0.05), we will use a time-dependent covariate model or stratified Cox model.” *(see Methods, Statistical analysis, lines 232-238)*

the potential for differential post-reversal propofol exposure between groups as a confounder;

Response: Thank you. We acknowledge the concern regarding potential confounding from differential post-reversal propofol exposure. To address this: (1) We have clarified that propofol sedation is standardized to BIS 60–80 until TOF ratio > 0.9 during the post-reversal period in both groups, minimizing discretionary differences: “Patients will be sedated with propofol infusion of 1–3 mg/kg/h to achieve a BIS of 60–80 until TOF ratio > 0.9.” *(see Methods, Study interventions, lines 134‒136)*

(2) We will record and compare cumulative propofol infusion dose between groups: “Cumulative propofol dose from study drug administration (reversal) to extubation will be recorded.” *(see Methods, Data collection and management, lines 184‒185)*

(3) Propofol exposure will be included as a covariate in sensitivity analysis to assess its confounding effect: “In addition, we will adjust for propofol exposure as a covariate in the sensitivity analysis of the primary endpoint.” *(see Methods, Statistical analysis, lines 230‒231)*

and the risk of differential outcome ascertainment due to variable discharge timing, necessitating pre-specified sensitivity analyses.

Response: Thank you for this important comment. We have acknowledged this potential ascertainment bias and clarified that this limitation will be addressed through analytical approaches: “The primary outcome window (within 7 postoperative days or until discharge) may lead to differential outcome ascertainment if discharge timing differs between groups. We will conduct a sensitivity landmark analysis restricted to patients with a minimum observation time of 72 hours (i.e., those remaining hospitalized for ≥ 3 days), ensuring comparable ascertainment windows between groups. In our pilot study, the median postoperative hospital stay was 7–10 days with only 2 patients (< 3%) discharged before postoperative day 3, indicating that this sensitivity analysis will retain > 97% of the cohort while effectively addressing potential ascertainment bias.” *(see Methods, Statistical analysis, lines 247‒255)*

The sample size calculation, while explicit, would benefit from a stronger justification for the assumed effect size on delirium.

Response: Thank you. We have clarified the sample size calculation and provided additional justification based on our internal pilot data: “We conservatively assumed a 12.5% absolute risk reduction (25% with sugammadex vs. 12.5% with neostigmine/glycopyrrolate), which is supported by our pilot trial conducted at the same center using identical 3D-CAM assessments. In that study, active interventions showed approximately 11% absolute reductions in POD incidence. This empirical evidence, combined with the anti-inflammatory and anti-oxidative properties of enhanced cholinergic signaling by neostigmine, supports the clinical plausibility of the assumed effect size.” *(see Methods, Sample size calculation, lines 203‒208)*

The plan should also address multiplicity given the numerous secondary outcomes, specify handling of clinical heterogeneity from the mixed surgical population, and provide more detail on quantitative neuromuscular monitoring.

Response: We appreciate these important methodological suggestions. We have clarified the multiplicity control for the secondary and exploratory endpoints: “To control for multiplicity, we will apply the Bonferroni correction for three secondary outcomes (days with POD, highest CAM-S score, and 30-day cognitive function), using a significance threshold of α = 0.017 (0.05/3). All exploratory outcomes (including pain scores, QoR-15 scores, non-delirium complications, 30-day mortality, and neuromuscular recovery metrics) will be analyzed using descriptive statistics only and interpreted as hypothesis-generating without adjustment for multiple comparisons.” *(see Methods, Statistical Analysis, lines 256‒262)*

While the primary objective is cognitive protection, we agree that documenting neuromuscular recovery is essential. We have added: “In addition, the neuromuscular recovery outcomes include time to TOF ratio > 0.9, incidence of residual neuromuscular blockade at extubation (TOF ratio < 0.9), time to extubation, and length of PACU stay.” *(see Methods, Study outcomes, lines 168‒171)*

Strengths include standardized anesthesia, rigorous masking, and proper registration. Minor revisions suggested for improved reproducibility and clarity involve detailing assessor training, specifying data sharing plans, and streamlining terminology.

Response: Thank you for this suggestion. We have added details regarding assessor training and inter-rater reliability in the Methods: “Postoperative follow-up will be performed by two independent outcome assessors, who will undergo standardized training prior to study initiation (including completion of the online 3D-CAM training and bedside practical training supervised by a board-certified neuropsychologist), as specified in our recent pilot study. Inter-rater reliability will be assessed during the initial phase of the trial (first 20 patients) using Cohen's kappa; an acceptable value of ≥ 0.80 will be required before formal recruitment proceeds.” *(see Methods, Data collection and management, lines 185‒192)*

We have clarified the data availability statement: “All de-identified individual participant data collected during the trial, study protocol, statistical analysis plan, and informed consent form, will be made available in the Mendeley Data at the time of publication. Identifiers (e.g., name, medical record number, exact date of birth, contact information) will be removed and replaced with unique study IDs.” *(see Data availability statement, lines 330‒334)*

We have ensured consistent use of “postoperative delirium (POD)” throughout the manuscript, with the full term defined only at first mention in the Abstract and Introduction, and the abbreviation “POD” used uniformly thereafter.

**Reviewers' comments:**

Reviewer's Responses to Questions

**Comments to the Author**

1. Does the manuscript provide a valid rationale for the proposed study, with clearly identified and justified research questions?

The research question outlined is expected to address a valid academic problem or topic and contribute to the base of knowledge in the field.

Reviewer #1: Yes

Reviewer #2: Yes

2. Is the protocol technically sound and planned in a manner that will lead to a meaningful outcome and allow testing the stated hypotheses?

The manuscript should describe the methods in sufficient detail to prevent undisclosed flexibility in the experimental procedure or analysis pipeline, including sufficient outcome-neutral conditions (e.g. necessary controls, absence of floor or ceiling effects) to test the proposed hypotheses and a statistical power analysis where applicable. As there may be aspects of the methodology and analysis which can only be refined once the work is undertaken, authors should outline potential assumptions and explicitly describe what aspects of the proposed analyses, if any, are exploratory.

Reviewer #1: Partly

Reviewer #2: Yes

3. Is the methodology feasible and described in sufficient detail to allow the work to be replicable?

Descriptions of methods and materials in the protocol should be reported in sufficient detail for another researcher to reproduce all experiments and analyses. The protocol should describe the appropriate controls, sample size calculations, and replication needed to ensure that the data are robust and reproducible.

Reviewer #1: Yes

Reviewer #2: Yes

4. Have the authors described where all data underlying the findings will be made available when the study is complete?

The [PLOS Data policy](https://journals.plos.org/plosone/s/materials-and-software-sharing) requires authors to make all data underlying the findings described in their manuscript fully available without restriction, with rare exception, at the time of publication. The data should be provided as part of the manuscript or its supporting information, or deposited to a public repository. For example, in addition to summary statistics, the data points behind means, medians and variance measures should be available. If there are restrictions on publicly sharing data—e.g. participant privacy or use of data from a third party—those must be specified.

Reviewer #1: Yes

Reviewer #2: Yes

5. Is the manuscript presented in an intelligible fashion and written in standard English?

PLOS ONE does not copyedit accepted manuscripts, so the language in submitted articles must be clear, correct, and unambiguous. Any typographical or grammatical errors should be corrected at revision, so please note any specific errors here.

Reviewer #1: Yes

Reviewer #2: Yes

6. Review Comments to the Author

Please use the space provided to explain your answers to the questions above and, if applicable, provide comments about issues authors must address before this protocol can be accepted for publication. You may also include additional comments for the author, including concerns about research or publication ethics.

You may also provide optional suggestions and comments to authors that they might find helpful in planning their study.

(Please upload your review as an attachment if it exceeds 20,000 characters)

**Reviewer #1:** This manuscript presents a well-structured study protocol for a single-center, triple-masked randomized controlled trial comparing neostigmine/glycopyrrolate with sugammadex for neuromuscular blockade reversal, with postoperative delirium (POD) as the primary outcome in older adults. The clinical rationale is relevant, the research question is clearly stated, and the protocol includes ethical approval and trial registration. Overall, the study has the potential to address an important perioperative question; however, several methodological and statistical aspects require clarification to strengthen internal validity and replicability.

**Major comments:**

The analysis population is described as including randomized patients with available outcome data, and no imputation is planned for missing data. This corresponds to a modified intention-to-treat approach and may introduce bias if POD assessments are missing non-randomly (e.g., early discharge or clinical deterioration). The authors should clearly define the primary analysis set (ITT vs modified ITT) and pre-specify how missing POD assessments will be handled, including consideration of sensitivity analyses.

Response: We appreciate the reviewer’s concern regarding potential bias from missing data. Importantly, our recently completed pilot trial (Long et al., Anesth Analg 2024, PMID: 37874773) employed an identical assessment protocol (twice-daily 3D-CAM evaluations by the same trained research team at our institution) in a comparable elderly surgical population (major non-cardiac surgery, mean age 69.6 years). In that study, POD assessments were completed without exception in all 78 randomized patients (100% ascertainment). Based on this, we expect minimal missing data (< 5%) in the current trial and have retained the complete case analysis as primary. Comprehensive sensitivity analyses will also be conducted to address any unforeseen missingness.

We have clarified this in the Statistical Analysis: “The primary analysis will be conducted in a modified intention-to-treat population, defined as all randomized patients who undergo surgery and have at least one postoperative POD assessment. In our recently completed pilot trial using identical assessment protocols, twice-daily 3D-CAM evaluations were completed without exception in all 78 older surgical patients (100% ascertainment). Based on this empirical evidence, we expect minimal missing data (< 5%) and will perform the primary analysis on a complete case basis. To assess robustness to any unforeseen missingness, we will conduct: (1) worst-case scenario analysis (all missing data assumed to be POD in the neostigmine/glycopyrrolate group and no POD in the sugammadex group); (2) best-case scenario analysis (reverse of worst-case); and (3) per-protocol analysis (excluding patients with major protocol deviations). If the observed missing data rate exceeds 10%, an additional sensitivity analysis using multiple imputation will be conducted.” *(see Methods, Statistical analysis, lines 218‒230)*

The statistical plan mentions Kaplan–Meier analysis and hazard ratios for time to POD, but does not specify the survival model, time origin, or how event time will be assigned given twice-daily assessments (with potential interval censoring). These elements should be explicitly defined, and assumptions (e.g., proportional hazards) should be addressed.

Response: Thank you for this helpful comment. We have clarified the assessment of POD, “In addition to the twice-daily POD assessments, a 12-h look-back data extraction from medical records and nursing notes will be performed to capture any CAM-positive signs that may have occurred within the preceding 12 hours. The day of the first positive 3D-CAM assessment (morning, evening, or 12-h look-back) is taken as the event date.” *(see Methods, Study outcomes, lines 147‒152)*

We have clarified the survival model, time origin, and assumptions in the Statistical Analysis: “Kaplan–Meier curves will be used to describe time to first POD, with the day of surgery set as day 0. Time-to-event analysis will be conducted on a discrete-time scale (calendar days). The treatment effect will be estimated with a Cox proportional-hazards model. As a sensitivity analysis for competing risks (death and early discharge), we will estimate cumulative incidence functions using the Fine-Gray model. If the proportional hazards assumption is violated (Schoenfeld residuals test *P* < 0.05), we will use a time-dependent covariate model or stratified Cox model.” *(see Methods, Statistical analysis, lines 232‒238)*

The primary outcome window (“within 7 postoperative days or until discharge”) may lead to differential outcome ascertainment if discharge timing differs between groups. The authors should clarify how this limitation will be addressed analytically and consider sensitivity analyses based on minimum observation time.

Response: Thank you for this important comment. We have acknowledged this potential ascertainment bias and clarified that this limitation will be addressed through analytical approaches: “The primary outcome window (within 7 postoperative days or until discharge) may lead to differential outcome ascertainment if discharge timing differs between groups. We will conduct a sensitivity landmark analysis restricted to patients with a minimum observation time of 72 hours (i.e., those remaining hospitalized for ≥ 3 days), ensuring comparable ascertainment windows between groups. In our pilot study, the median postoperative hospital stay was 7–10 days with only 2 patients (< 3%) discharged before postoperative day 3, indicating that this sensitivity analysis will retain > 97% of the cohort while effectively addressing potential ascertainment bias.” *(see Methods, Statistical analysis, lines 247‒255)*

**Minor comments:**

Additional details on assessor training and any assessment of inter-rater reliability for POD diagnosis would improve reproducibility.

Response: Thank you for this suggestion. We have added details regarding assessor training and inter-rater reliability in the Methods: “Postoperative follow-up will be performed by two independent outcome assessors, who will undergo standardized training prior to study initiation (including completion of the online 3D-CAM training and bedside practical training supervised by a board-certified neuropsychologist), as specified in our recent pilot study. Inter-rater reliability will be assessed during the initial phase of the trial (first 20 patients) using Cohen's kappa; an acceptable value of ≥ 0.80 will be required before formal recruitment proceeds.” *(see Methods, Data collection and management, lines 185‒192)*

Clarification on whether MMSE scores are adjusted for education or language norms would strengthen baseline cognitive characterization.

Response: Thank you. We have clarified the education-adjusted MMSE scoring in the Methods: “Baseline cognitive function will be assessed with the 30-point Chinese version of the Mini-Mental State Examination (MMSE), covering six domains: orientation to time and place, word repetition, attention and calculation, language expression, speech comprehension, and simple motor commands. Education-adjusted cutoff values for cognitive impairment will be applied: < 24 for patients with postsecondary education, < 23 for secondary education, < 20 for primary education, and < 18 for illiterate patients.” *(see Methods, Data collection and management, lines 176‒182)*

The data availability statement could be made more specific by indicating the intended repository, de-identification process, and timing of data release.

Response: Thank you. We have clarified the data availability statement: “All de-identified individual participant data collected during the trial, study protocol, statistical analysis plan, and informed consent form, will be made available in the Mendeley Data at the time of publication. Identifiers (e.g., name, medical record number, exact date of birth, contact information) will be removed and replaced with unique study IDs.” *(see Data availability statement, lines 330‒334)*

Addressing these points would improve transparency and reduce analytical flexibility, thereby strengthening the protocol’s ability to test the stated hypothesis.

Response: Thank you again for your time and helpful suggestions.

**Reviewer #2:** This protocol describes a well-designed, triple-masked, randomized trial that addresses an important, clinically relevant question. The primary endpoint and sample size calculation are clearly specified and methodologically appropriate. The study's strengths include rigorous blinding, validated delirium assessments, and trial registration. However, the expected effect size may be overly optimistic, and the heterogeneity of surgical populations warrants further consideration. The protocol would be strengthened by clarifying multiplicity handling and analytical details.

**MAJOR COMMENTS**
Clarity and Appropriateness of Endpoints:
The primary endpoint, the incidence of postoperative delirium within seven days or until discharge (assessed twice daily using the 3D-CAM), is clearly defined, clinically relevant, and based on a validated instrument.
Secondary and exploratory endpoints are clearly listed. However, the manuscript includes a large number of these outcomes, which increases the risk of multiplicity and selective emphasis. The authors should clarify which secondary outcomes are considered key and explain how they will handle multiplicity (e.g., hierarchical testing or interpretation as hypothesis-generating).
No information is provided about the effect of neostigmine and sugammadex on neuromuscular block reversal. This could be another important secondary endpoint that most readers would expect.

Response: We appreciate these important methodological suggestions. We have clarified the multiplicity control for the secondary and exploratory endpoints: “To control for multiplicity, we will apply the Bonferroni correction for three secondary outcomes (days with POD, highest CAM-S score, and 30-day cognitive function), using a significance threshold of α = 0.017 (0.05/3). All exploratory outcomes (including pain scores, QoR-15 scores, non-delirium complications, 30-day mortality, and neuromuscular recovery metrics) will be analyzed using descriptive statistics only and interpreted as hypothesis-generating without adjustment for multiple comparisons.” *(see Methods, Statistical Analysis, lines 256‒262)*

While the primary objective is cognitive protection, we agree that documenting neuromuscular recovery is essential. We have added: “In addition, the neuromuscular recovery outcomes include time to TOF ratio > 0.9, incidence of residual neuromuscular blockade at extubation (TOF ratio < 0.9), time to extubation, and length of PACU stay.” *(see Methods, Study outcomes, lines 168‒171)*

Consistency between endpoints and sample size calculation:
The sample size calculation is explicitly based on the primary endpoint (POCD in the first 7 days) and is methodologically sound, with clearly stated assumptions (baseline POD incidence, expected absolute risk reduction, α, and power).
However, the assumed 12.5% absolute risk reduction is derived from early cognitive dysfunction (first POD) rather than POD itself. The authors should better justify the clinical plausibility of this effect size for delirium or acknowledge the risk of overestimating the expected treatment effect.

Response: We appreciate this concern regarding the assumed effect size. We have clarified the sample size calculation and provided additional justification based on our internal pilot data: “We conservatively assumed a 12.5% absolute risk reduction (25% with sugammadex vs. 12.5% with neostigmine/glycopyrrolate), which is supported by our pilot trial conducted at the same center using identical 3D-CAM assessments. In that study, active interventions showed approximately 11% absolute reductions in POD incidence. This empirical evidence, combined with the anti-inflammatory and anti-oxidative properties of enhanced cholinergic signaling by neostigmine, supports the clinical plausibility of the assumed effect size.” *(see Methods, Sample size calculation, lines 203‒208)*

The randomized, triple-masked design with standardized anesthetic management and quantitative neuromuscular monitoring is a major strength.
Nonetheless, the inclusion of a heterogeneous surgical population (thoracic, abdominal, orthopedic, spinal, and urologic surgeries) may dilute the effect size and introduce clinical heterogeneity that could affect POD incidence. This issue should be discussed more explicitly, and stratification or interaction analyses should be specified more clearly in advance.

Response: We appreciate this important comment. We have clarified the interaction tests in the Statistical Analysis: “Subgroup analyses of the POD incidence will be conducted according to age (65‒80 years or > 80 years), sex (male or female), ASA classification (I‒II or III), preoperative MMSE (< 23 or ≥ 23), and type of surgery (thoracic, abdominal, orthopedic/spinal, urologic). We will perform interaction tests (treatment-by-surgical-type) to assess whether the treatment effect on POD differs significantly across surgical subspecialties. If significant heterogeneity is detected (*P* for interaction < 0.10), we will report stratum-specific estimates; otherwise, the pooled estimate will be presented as the primary result.” *(see Methods, Statistical Analysis, lines 239‒246)*

In the Discussion, we have added: “Last, the inclusion of a heterogeneous surgical population (thoracic, abdominal, orthopedic, spinal, and urologic surgeries) may dilute the effect size and introduce clinical heterogeneity that could affect POD incidence; however, we intend to enhance the generalizability of findings across the broad spectrum of major non-cardiac surgery to reflect real-world clinical practice where reversal agents are used uniformly regardless of surgical type. Pre-specified subgroup analyses can mitigate this concern. Our pilot study was also conducted in the same surgical population” *(see Discussion, 312‒319)*

Planned Statistical Analysis:
The statistical plan is generally appropriate for the primary endpoint.
The use of the Kaplan-Meier method to analyze time to POD should be more clearly justified, especially with regard to how discharges before day 7 will be handled analytically (competing risk versus censoring).
No plan for handling missing data is provided beyond stating that no imputation will be used, which may be problematic in an elderly population with repeated assessments.

Response: Thank you. We have clarified the Kaplan-Meier method to analyze time to POD, including the survival model, time origin, and assumptions: “Kaplan–Meier curves will be used to describe time to first POD, with the day of surgery set as day 0. Time-to-event analysis will be conducted on a discrete-time scale (calendar days). The treatment effect will be estimated with a Cox proportional-hazards model. As a sensitivity analysis for competing risks (death and early discharge), we will estimate cumulative incidence functions using the Fine-Gray model. If the proportional hazards assumption is violated (Schoenfeld residuals test *P* < 0.05), we will use a time-dependent covariate model or stratified Cox model.” *(see Methods, Statistical analysis, lines 232-238)*

We appreciate the reviewer’s concern regarding potential bias from missing data. Importantly, our recently completed pilot trial (Long et al., Anesth Analg 2024, PMID: 37874773) employed an identical assessment protocol (twice-daily 3D-CAM evaluations by the same trained research team at our institution) in a comparable elderly surgical population (major noncardiac surgery, mean age 69.6 years). In that study, POD assessments were completed without exception in all 78 randomized patients (100% ascertainment). Based on this, we expect minimal missing data (< 5%) in the current trial and have retained the complete case analysis as primary. Comprehensive sensitivity analyses will also be conducted to address any unforeseen missingness.

We have clarified this in the Statistical Analysis: “The primary analysis will be conducted in a modified intention-to-treat population, defined as all randomized patients who undergo surgery and have at least one postoperative POD assessment. In our recently completed pilot trial using identical assessment protocols, twice-daily 3D-CAM evaluations were completed without exception in all 78 older surgical patients (100% ascertainment). Based on this empirical evidence, we expect minimal missing data (< 5%) and will perform the primary analysis on a complete case basis. To assess robustness to any unforeseen missingness, we will conduct: (1) worst-case scenario analysis (all missing data assumed to be POD in the neostigmine/glycopyrrolate group and no POD in the sugammadex group); (2) best-case scenario analysis (reverse of worst-case); and (3) per-protocol analysis (excluding patients with major protocol deviations). If the observed missing data rate exceeds 10%, an additional sensitivity analysis using multiple imputation will be conducted.” *(see Methods, Statistical analysis, lines 218‒230)*

Discussion and Interpretation:
As this is a protocol, the discussion appropriately focuses on rationale and mechanisms. However, some statements anticipate benefits and clinical impacts that may appear overly optimistic for a superiority trial whose results are not yet known. The discussion should more explicitly acknowledge uncertainty and the possibility of neutral or negative findings.

Response: Thank you. We have revised the Discussion to more explicitly acknowledge uncertainty. In the Discussion, we have added explicit statements regarding the possibility of non-significant findings: “However, if the true effect size is smaller than the assumed 12.5% absolute risk reduction, or if biological heterogeneity increases variance, the findings may be null or negative. Such a finding would suggest that the observed cholinergic effects of neostigmine do not translate into clinically meaningful cognitive protection in the broad surgical population. Given the biological complexity of POD, it is possible that the treatment effect may be heterogeneous across subgroups, and the overall trial results may mask beneficial effects in specific phenotypes that would warrant investigation in future studies.” *(see Discussion, lines 297‒304)*

Ethics Approval and Trial Registration:
Ethics approval is clearly reported with the committee name and approval number. Trial registration in the Chinese Clinical Trial Registry is appropriately reported, including the registration number and the date prior to patient enrollment.
These aspects are adequately addressed and compliant with international standards.

Response: We thank the reviewer for confirming that the ethics approval and trial registration information meets international standards.

**MINOR COMMENTS**
Terminology: Ensure consistent use of "postoperative delirium (POD)" throughout the manuscript. Avoid repeating full definitions once they have been established.

Response: We thank the reviewer for this suggestion. We have ensured consistent use of “postoperative delirium (POD)” throughout the manuscript, with the full term defined only at first mention in the Abstract and Introduction, and the abbreviation “POD” used uniformly thereafter.

Exploratory Outcomes: The long list of exploratory outcomes (e.g., pain, QoR-15, complications, and mortality) could be streamlined or clearly framed as descriptive only.

Response: Thank you. We have clarified in the Statistical Analysis section: “All exploratory outcomes (including pain scores, QoR-15 scores, non-delirium complications, 30-day mortality, and neuromuscular recovery metrics) will be analyzed using descriptive statistics only and interpreted as hypothesis-generating without adjustment for multiple comparisons.” *(see Methods, Statistical analysis, lines 258‒262)*

Generalizability: Excluding ICU patients and cardiac surgery patients limits the study's external validity, and this should be more clearly acknowledged as a design choice.

Response: Thank you. We have added this point in the limitations: “The exclusion of cardiac surgery patients and patients requiring postoperative ICU admission limits the external validity of our findings to these high-risk populations, where the pathophysiology of delirium may differ (e.g., involving systemic inflammation induced by cardiopulmonary bypass or encephalopathy in critical illness). It is our design choice to isolate the specific effects of reversal agents from these confounding factors.” *(see Discussion, lines 307‒312)*

A brief description of TOF technical data is warranted, including the type of monitoring device, whether the calibration protocol is prespecified, and the choice of monitoring site.

Response: Thank you. We have clarified the details of TOF monitoring: “TOF monitoring will be performed using TOF-Watch SX at the adductor pollicis muscle via ulnar nerve stimulation at the wrist. We will follow a prespecified calibration protocol: after induction and before rocuronium administration, the device is calibrated to achieve supramaximal stimulation and a stable baseline TOF ratio > 0.9; calibration will be repeated until three consecutive readings vary by < 5%.” *(see Methods, Anesthetic care, lines 111‒116)*

7. PLOS authors have the option to publish the peer review history of their article ([what does this mean?](https://journals.plos.org/plosone/s/editorial-and-peer-review-process#loc-peer-review-history)). If published, this will include your full peer review and any attached files.

If you choose “no”, your identity will remain anonymous but your review may still be made public.

**Do you want your identity to be public for this peer review?** For information about this choice, including consent withdrawal, please see our [Privacy Policy](https://www.plos.org/privacy-policy).

Reviewer #1: **Yes:** Yahya Kayed AbuJwaid

Reviewer #2: **Yes:** Federico Piccioni
