# Supplementary material for: Effect of neostigmine/glycopyrrolate versus sugammadex on postoperative delirium in older adults: A triple-masked, randomized, controlled trial protocol
Source: PLoS One. 2026 Apr 1;21(4):e0346523. doi: 10.1371/journal.pone.0346523 (PMC13042718; doi:10.1371/journal.pone.0346523)
Supplement: Attachment — Submitted filename: Response_to_Reviewers_auresp_2.docx [file pone.0346523.s006.docx]

1. The missing data assumption posits that, while minimal missingness is anticipated, it may be beneficial to explicitly articulate the assumption underlying the primary complete-case analysis (e.g., missing completely at random) and briefly acknowledge this in the interpretation framework.

Thank you. We have now explicitly stated in the Statistical analysis section that the primary complete-case analysis assumes data are missing completely at random, given the anticipated minimal missingness (< 5%) based on our pilot trial experience. *(see page 10, lines 232‒233)*

We have also added a brief acknowledgment in the Discussion regarding this assumption and its potential limitation. *(see page 15, lines 336‒340)*

2. The time-to-event approximation is determined as follows: In the context of twice-daily POD assessments, event timing is interval-censored within a 12-hour window. A concise acknowledgment that the discrete-time approach constitutes an approximation would enhance methodological transparency.

Thank you. We have now explicitly acknowledged in the Statistical analysis section that the discrete-time approach for time-to-event analysis constitutes an approximation, given that the twice-daily POD assessments result in interval-censored event timing within a 12-hour window. *(see page 10, lines 242‒243)*

3. The proposed plan to adjust for cumulative propofol exposure is deemed to be appropriate. It is imperative to consider the specific regression framework in which this covariate will be incorporated.

Thank you. We have now clarified that cumulative propofol exposure will be incorporated as a continuous covariate in the logistic regression model for the primary binary outcome and in the Cox proportional-hazards model for time-to-event analysis, with effect estimates reported as adjusted odds ratio and hazard ratio, respectively. *(see page 11, lines 248‒252)*

4. The assumption of effect size is predicated on the premise that, while substantiated by preliminary data, the anticipated absolute risk reduction of 12.5% may in fact prove to be overly optimistic. A brief acknowledgment that a smaller true effect could result in reduced power would serve to strengthen balance in the protocol.

Thank you. We have now added a brief acknowledgment in the Sample size calculation section that the assumed 12.5% absolute risk reduction, while supported by preliminary data and mechanistic rationale, may prove optimistic, and that a smaller true effect would result in reduced statistical power. *(see page 9, lines 214‒217)*

5. Multiplicity and secondary outcomes – The Bonferroni approach is deemed appropriate. It is imperative to elucidate that secondary analyses will persist in their capacity to provide supportive evidence and to generate hypotheses, a state of affairs that may engender enhanced interpretive consistency.

Thank you. We have now clarified in the Statistical analysis section that secondary analyses will serve a supportive role for the primary outcome and help generate hypotheses for future research, ensuring balanced interpretation of findings. *(see page 12, lines 274‒276)*

6. Subgroup Analyses
As multiple subgroup and interaction analyses have been predetermined, elucidating their exploratory nature (absent a substantial interaction detection) would prove beneficial.

Thank you. We have now explicitly stated in the Statistical analysis section that all subgroup and interaction analyses are exploratory in nature unless a substantial interaction is detected, to prevent overinterpretation of subgroup findings. *(see page 11, lines 258‒260)*

7. Neuromuscular Monitoring – The inclusion of supplementary technical details is appreciated. A brief statement confirming adherence to current international recommendations for quantitative neuromuscular monitoring would further strengthen reproducibility.

Thank you. We have now added a brief statement in the Anesthetic care section confirming that our neuromuscular monitoring protocol adheres to current international recommendations for quantitative monitoring (ESAIC 2023 and ASA 2023 guidelines), ensuring standardized calibration and assessment of neuromuscular block and recovery. *(see page 5, lines 116‒120)*
